# Extracellular Matrix Characterization in Gastric Cancer Helps to Predict Prognosis and Chemotherapy Response

**DOI:** 10.3389/fonc.2021.753330

**Published:** 2021-09-27

**Authors:** Zhi Yang, Feifei Xue, Minhuan Li, Xingya Zhu, Xiaofeng Lu, Chao Wang, En Xu, Xingzhou Wang, Liang Zhang, Heng Yu, Chuanfu Ren, Hao Wang, Yizhou Wang, Jie Chen, Wenxian Guan, Xuefeng Xia

**Affiliations:** ^1^ Department of General Surgery, Drum Tower Clinical Medical College of Nanjing Medical University, Nanjing, China; ^2^ Jiangsu Key Laboratory of Oral Diseases, Nanjing Medical University, Nanjing, China; ^3^ Department of Andrology, Drum Tower Clinical Medical College of Nanjing Medical University, Nanjing, China; ^4^ Department of General Surgery, Nanjing Drum Tower Hospital, The Affiliated Hospital of Nanjing University Medical School, Nanjing, China; ^5^ Department of Anesthesiology, Nanjing Drum Tower Hospital, The Affiliated Hospital of Nanjing University Medical School, Nanjing, China

**Keywords:** gastric cancer, ECM, prognostic factors, chemotherapy response, multi-omics

## Abstract

The extracellular matrix (ECM) plays a central role in the formation of the tumor microenvironment. The deposition of the ECM is associated with poor prognosis in a variety of tumors. Aberrant ECM deposition could undermine the effect of chemotherapy and immunotherapy. However, there is no systematic analysis on the relationship between the ECM and prognosis or chemotherapy effect. In the present study, we applied the gene set variation analysis (GSVA) algorithm to score 2199 canonical pathways in 2125 cases of probe or sequencing data and identified the core matrisome as the driving factor in gastric cancer progression. We classified gastric cancer samples into three clusters according to the composition of the ECM and evaluated clinical and multi-omics characterization of ECM phenotypes. The ECM score was evaluated by GSVA score of core matrisome and a higher ECM score predicted poor prognosis of gastric cancer [Hazard Ratio (HR), 2.084; p-value < 2 × 10^−16^]. In The Cancer Genome Atlas (TCGA) cohort and KUGH, YUSH, and KUCM cohorts, we verified that patients with a low ECM score could benefit from chemotherapy. By contrast, patients with a high ECM score did not achieve satisfactory response from chemotherapy. Determining the characteristics of the ECM microenvironment might help to predict the prognosis and chemotherapy response of patients with gastric cancer, and help to resolve the enigma of chemoresistance acquisition, as well as providing inspiration to develop combination therapy.

## Introduction

Gastric cancer (GC) is the third leading cause of cancer-related death and the fifth most common cancer diagnosed worldwide (1). Surgical resection has always been the mandatory backbone treatment for resectable stage II and III GC (1). However, the significant benefit from surgical resection alone is confined to early GC, while the rate of relapse remains high for advanced GC.

Multimodal therapies, including chemotherapy, chemoradiation, and immunotherapy, have been established to prevent recurrence and have improved the survival rates of patients after surgery (1). Although the receipt of adjunctive therapies could improve prognosis for some patients with GC patients to a certain extent, variations in clinical outcome have been detected for patients who received the same treatment (2–4). Multiple molecular subtypes and ingenious prognostic models based on multi-omics data have been established for patients with resectable GC. Stomach adenocarcinoma in The Cancer Genome Atlas (TCGA) was subdivided into five molecular subtypes on the basis of molecular profiles: microsatellite instable (MSI), genomically stable (GS), Epstein–Barr virus (EBV) associated, chromosomal instability (CIN) and hypermutated-single-nucleotide variant predominant (HM-SNV) (5, 6). And the Asian Cancer Research Group (ACRG) defined four molecular subtypes, including microsatellite stable (MSS)/epithelial–mesenchymal transition (EMT), MSI, MSS/p53^+^, and MSS/p53^−^ (7). Oh et al. (8)identified two distinct molecular subtypes: mesenchymal phenotype(MP) and epithelial phenotype (EP). These molecular subtypes show great tumor heterogeneity, distinct clinical outcome and different response to anti-tumor therapy. Additionally, Zeng et al. (9) depicted the comprehensive landscape of tumor microenvironment characteristics and established TMEscore based on tumor immune infiltration patterns to predict immunotherapy response in gastric cancer. Zhang et al. (10) characterized m^6^A modification patterns in gastric cancer and constructed m6Ascore based on 21 m^6^A regulators, which could also discriminate distinct TME and do well in predicting benefits from immunotherapy for patients with gastric cancer. Cheong et al. (11) developed and validated a model with four classifier genes (GZMB, WARS, SFRP4, and CDX1) for predicting adjuvant chemotherapy response in patients with resectable, stage II–III gastric cancer. Benefited from these classification and scoring system, the tumor heterogeneity could be defined, evaluated and precisely targeted.

The extracellular matrix (ECM) regulates tissue development and homeostasis (12). It consists of biochemically and biomechanically distinct macromolecules, including glycoproteins, collagens, and proteoglycans, which assemble into a three-dimensional supramolecular network that regulates cell growth, survival, motility, and differentiation (13). As a major component of the tumor microenvironment, the ECM could affect the hallmarks of cancer and is involved in all the cellular processes contributing to cancer initiation, progression, and dissemination (14, 15). Researchers found that increased ECM stiffness is required for the transformation of normal cells into tumor through YAP/TAZ mechanotransduction (16), and could also drive EMT, invasion and metastasis *via* TWIST1–G3BP2 mechanotransduction (17). In gastric cancer, the stiffness of the ECM could induce hypomethylation of the promoter region of mechanosensitive Yes-associated protein (YAP) and activate the oncogenic activity of YAP (18). Clinical observations also confirmed that an increased ECM content correlates with more aggressive tumors and poorer prognosis (Socovich and Naba 2019). Intriguingly, in pancreatic ductal adenocarcinoma, decreasing ECM with an anti–lysyl oxidase like-2 (anti-LOXL2) antibody in syngeneic orthotopic PDA mouse models accelerated tumor growth, resulting in diminished overall survival, which suggested a protective role of ECM (20). In addition, tumor ECM is also an affecting factor of cancer therapy. A pan-cancer analysis showed that ECM deposition induced by TGF-β signalling could predict failure of PD-1 blockade (21). On the contrary, inhibiting ECM deposition could soften metastases of colorectal cancer and increase the anti-angiogenic effects of bevacizumab (22). However, until recently, we were not aware of the whole picture of the complexity of the tumor ECM, nor had we determined, to what extent, the ECM is involved in cancer progression. Rapidly developing high throughput sequencing and bioinformatic technologies are of great help to precisely characterize the ECM composition in tumor microenvironments. In this study, we are going to characterize the landscape of ECM in gastric cancer and discuss its clinical implications.

## Materials and Methods

### Gene Expression Data Gathering and Processing

We searched in The Cancer Genome Atlas (TCGA) database and Gene-Expression Omnibus (GEO) for open source gene-expression data with full clinical annotation of gastric cancer. Only those with a sample size greater than 50 and available survival information were included for further analysis. In total, 2125 gastric cancer samples were integrated, including 7 cohorts from the GEO database (https://www.ncbi.nlm.nih.gov/; geo accession numbers: GSE13861 [Yonsei University Severance Hospital (YUSH) cohort], GSE15459, GSE26253, GSE26942, GSE29272, GSE66229, GSE84437 [Asian Cancer Research Group (ACRG) cohort] and TCGA-STAD cohort (**Supplementary Table S1**). The GSE26942 cohort was merged with GSE26899 for the Korea University Guro Hospital (KUGH) cohort and with GSE26901 for the Kosin University College of Medicine (KUCM) cohort. In brief, primary microarray data sets downloaded from GEO were analyzed with background adjustment and normalized using the microarray data package in the R language environment (23). For the TCGA-STAD cohort, latest RNA-sequencing data (HTSeq-FPKM) were retrieved through the R package TCGAbiolinks 2.16.4, which was further transformed into transcripts per kilobase million (TPM) to make it more comparable with the microarray data. All the gene expression data sets were transformed into a log2 base before further analysis. To merge multiple gene expression data sets, a batch normalization algorithm was employed to remove batch effects using the R package sva 3.36.0.

### Clinical and Genomic Data Collection

Up to date clinical information for the TCGA-STAD cohort was downloaded and prepared using the R package TCGAbiolinks 2.16.4 and that of other cohorts was directly downloaded as attached files from GEO database or from the **Supplementary Materials** in the related literature. Multi-omics data of the TCGA-STAD cohort, including somatic mutation, copy number variation (CNV), and DNA methylation (Illumina Human Methylation 450K), were obtained from UCSC Xena (https://xenabrowser.net/). All the multi-omics data analysis was limited to samples with available mRNA data; therefore, we analyzed 366 samples for somatic mutation, 374 samples for CNVs, and 336 samples for DNA methylation.

### Gene Set Variation Analysis

We downloaded 2922 canonical pathways gene sets integrated from authoritative pathway databases, including the BioCarta pathway database, the Kyoto Encyclopedia of Genes and Genomes (KEGG) pathway database, the PID pathway database, the Reactome pathway database, and the WikiPathways pathway database, from the Molecular Signatures Database (MSigDB, https://www.gsea-msigdb.org/gsea/index.jsp) (24). The normalized GSVA score of each canonical pathway gene set was measured for each gastric cancer sample using the GSVA algorithm in the R package GSVA 1.36.2 (25). The ECM score was measured as the GSVA score of the core matrisome gene set downloaded from MatrisomeDB (http://www.pepchem.org/matrisomedb), an updated version with slight changes (26)

### Gene Set Enrichment Analysis

The GSEA algorithm was used to analyze the enriched biological processes between different groups. In brief, the differential genes between two groups were measured with the R package limma 3.44.3, and were subsequently pre-ranked by log2 fold-change and submitted to the R package clusterProfiler 3.16.1 to run the GSEA. Results with a p-value < 0.05 and a q-value < 0.05 were considered statistically significant.

### Consensus Clustering for the Extracellular Matrix Composition

To identify different ECM composition patterns and classify patients into distinct groups for further analysis, Unsupervised clustering analysis (based on the Euclidean distance and Ward’s linkage) was carried out based on the expression of 274 kinds of ECM in the merged data set and the ACRG cohort. The R package ConsensuClusterPlus 1.52.0 was used to perform the clustering procedure and to determine the optimal number of clusters, which was repeated 1000 times to guarantee the stability of classification.

### Estimation Tumor Microenvironment Cell Infiltration

To explore the immune cell infiltration composition of different ECM clusters, the CIBERSORT algorithm was used to analyze the proportions of 22 types of immune cells in each sample of the ACRG cohort using the R package CIBERSORT (27). CIBERSORT employed a deconvolution algorithm, along with support vector regression, to work on 547 specific immune cell-related genes and deconstructed 22 main types of immune cells, including CD8^+^ T cells, regulatory T cells (Tregs), M0 macrophages, M1 macrophages, and M2 macrophages. 1,000 permutations were performed to achieve robust quantification of the relative abundance of each infiltrated immune cell.

### Gene Silencing by Small Interfering RNA Transfection

AGS and Hs746T cells were seeded in 6-well plates at 2 × 10^5^ cell per well overnight, and then treated with 2 µg of negative control small interfering RNA (siRNA), FBN1-siRNA (targeting FBN1 encoding fibrillin 1) and LAMC1-siRNA (targeting LAMC1 encoding laminin subunit gamma 1) constructed by Shanghai GenePharma Company (Shanghai, China) along with 5 μL siRNA interferin reagent (Polyplus, New York, NY, USA). After incubation at 37°C with 5% CO_2_ for 48 h, the efficiency of gene silencing was determined using qRT-PCR.

### Apoptosis Assay

For the apoptosis assay, cells were seeded into a six-well plate and subjected to different treatments. The cell apoptosis assay was operated according to the manual of the fluorescein isothiocyanate (FITC) Annexin V Apoptosis Detection Kit (BD Biosciences, San Jose, CA, USA). The results were analyzed using FlowJo 10 software (FlowJo, Ashland, OR, USA).

### Transwell Invasion Assay

For the Transwell invasion assay, 5 × 10^4^ cells in a volume of 200 μL of serum-free medium were added into a Transwell chamber containing a polycarbonate membrane with 8.0 μm pores (353097; BD Falcon) and covered with a layer of Matrigel matrix (56234; Corning Inc., Corning, NY, USA). The chamber was then placed in a 24-well plate containing 600 μL of medium with 10% fetal bovine serum and incubated at 37°C with 5% CO_2_. After 24 h of incubation, non-migrated cells were wiped away and the remaining cells that had migrated through the bottom of the chamber were fixed in 4% paraformaldehyde followed by crystal violet staining and counting under a microscope.

### Statistical Analysis

Unpaired Student t tests and Wilcoxon rank-sum tests were used to evaluate the statistical significance of normally distributed and non-normally distributed variables, respectively, when comparing two groups. Kruskal-Wallis tests and One-way analysis of variance (ANOVA) was used to conduct difference comparisons of more than two groups (28). Spearman and distance correlational analyses were conducted using the R package Hmisc 4.4.1. Objects with a Spearman correlation coefficient greater than 0.5 were deemed strongly correlated (29). The hazard ratios (HR) of all prognostic factors was calculated using a univariate Cox proportional hazards regression model. The “surv_cutpoint” function of the R package survminer 0.4.8 was used to estimate the best cut off point for prognostic factors according to their relationship with the patients’ survival probability with the maximum rank statistic. For the ECM score, patients were then divided into ECM score low and ECM high groups according to the best cut off point. Then, survival curves were drawn using the Kaplan–Meier method. The statistical significance of the difference in survival probability was estimated using the log-rank test. The R package forestplot 1.10 was used to show the univariate prognosis analyses of different groups of prognostic factors. The visualization of ECM clusters was facilitated by the R package umap 0.2.6., to perform dimensionality reduction. The networks of canonical signaling pathways were constructed using the software Cytoscape 3.7.2 and the hub pathway was estimated by the Cytoscape plug-in CytoHubba. The R package ComplexHeatmap 2.4.3 generated all the heat maps. A Waterfall Chart was used to exhibit the overview of gene mutation landscape, which was generated the R package maftools 2.4.12. The statistical difference of CNVs between the ECM low and ECM high group was determined using the R package cnvaq 0.1.3. Then, the IGV 2.8.2 software was employed to visualize the CNV landscape of the two groups.

All the above analyses were performed using the R 4.0.0 software. All the statistical analyses were two-sided and a p-value < 0.05 was considered statistically significant.

## Results

### Identification of the Core Matrisome as the Major Factor Involved in Gastric Cancer Progression

**Figure 1:**
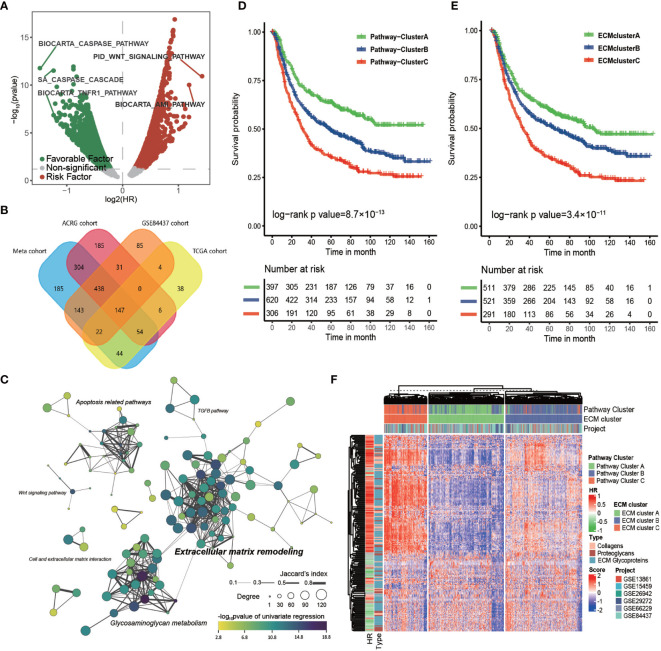
The core matrisome is major pathway involved in gastric cancer progression. **(A)** Volcano plot of prognostic pathways. The x-axis shows the log2 transformed hazard ratio and the y-axis shows −log10 transformed log rank p-values. Red dots indicate risk pathways; green dots indicate favorable pathways; and Grey dots indicate non-prognostic pathways. **(B)** Venn plot of the prognostic pathways in the TCGA-STAD cohort, ACRG cohort, GSE84437 cohort, and the meta-cohort. **(C)** Network of prognostic pathways in the meta-cohort. Each dot represents a prognostic pathway in gastric cancer. A line connecting two pathways means the Jaccard’s index between gene sets of the two pathways. The size of the dot represents the degree of the pathway in correlation network of these pathways. The color filling the dot shows the −log10 transformed p-value of univariate cox regression for the GSVA score of the corresponding gene set. **(D)** Kaplan–Meier curves for the overall survival of 1323 patients in the meta-cohort with 3 distinct pathway clusters. The sample size of pathway clusters A, B, and C were n = 397, n = 620, and n = 306, respectively. Log-rank test, p -value = 8.7 × 10^−13^. **(E)** Kaplan–Meier curves for overall survival of 1323 patients in the meta-cohort with 3 distinct ECM clusters. The sample size of ECM clusters A, B, and C were n = 511, n = 521, and n = 291, respectively. Log-rank test, p-value = 3.4 × 10^−11^. **(F)** Heat map showing the unsupervised clustering of 274 types of ECM for 1323 patients in the meta-cohort. The ECM clustering resembled the pathway clustering to a great extent. The hazard ratio and subtypes of ECM in the meta cohort are also shown in annotation on the right.

In total, eight eligible GC cohorts (GSE13861, GSE15459, GSE26253, GSE26942, GSE29272, ACRG/GSE66229, GSE84437, and TCGA-STAD) were used in our study. Six cohorts with microarray data (GSE13861, GSE15459, GSE26942, GSE29272, ACRG/GSE66229, and GSE84437) were merged into a meta-cohort (n = 1323). GSE26253 was not merged because of its limited number of gene probes and the sequence data of TCGA-STAD cohort was dismissed because it was being incompatible with the microarray data for technical reasons. Then, we calculated the GSVA score of 2199 canonical pathways for all the cohorts engaged in our study. First, the hazard ratio (HR) of the canonical pathways for overall survival (OS) of patients with gastric cancer were calculated in the meta-cohort (**Figure 1A**). The Wnt signaling pathway, the common pathway of fibrin clot formation, and autophagy were among the top risk pathways, and the caspase pathway, tumor necrosis factor receptor 1 (TNFR1) pathway, and Fas signaling pathway were among the top favorable pathways (**Supplementary Figure S1B**)

Then, we computed the HR of all the canonical pathways for four cohorts with more than 300 tumor samples (TCGA-STAD, ACRG/GSE66229, GSE84437, and meta-cohort; GSE26253 was not engaged because of its limited number gene probes). The intersection of those pathways, 147 in total, were considered to correlate stably with patients’ prognosis (**Figure 1B** and **Supplementary Table S2**). These 147 common pathways were further used to perform unsupervised hierarchical clustering for 1323 tumor samples in the meta-cohort. The result showed that these samples could be divided into three distinct subclusters, which displayed significant differences in survival (log-rank test, p-value < 0.001; **Figure 1D**).

To depict the biological processes that characterized the three pathway clusters, we performed GSEA for each cluster against the whole meta-cohort. The results showed that the top 10 gene ontology (GO) biological processes enriched in pathway cluster A primarily correlated with extracellular matrix organization and those of pathway cluster C were primarily correlated with mitosis and DNA replication (**Supplementary Figures S1C, D**), suggesting the importance of these pathways in gastric cancer progression. No pathway was enriched in pathway cluster B, implying it was intermediate between pathway cluster A and C.

To identify the core pathway involved in the OS of patients with gastric cancer, we calculated the Spearman correlation coefficient among the 147 common pathways in the four cohorts that had more than 300 tumor samples (**Supplementary Table S3**). Only those pathways with absolute value of the Spearman correlation coefficient greater than 0.5 were considered strongly correlated. The connectivity of each pathway in the correlation network was estimated for the four cohorts. Then, we constructed the clustering network of prognostic pathways for the meta cohort according to the intersection of these gene sets (**Figure 1C**). It showed that the most enriched pathways were extracellular matrix remodeling related pathways and glycosaminoglycan metabolism related pathways with higher degree and more closely related to prognosis followed by apoptosis related pathways. Moreover, the top 10 pathways ranked by degree for each cohort were estimated (**Supplementary Figures S1E–H**). The top four pathways with highest mean degree among the four cohorts were the core matrisome, ECM glycoproteins, ECM proteoglycans, and elastic fiber formation, all of which were included in the top 10 pathways in each of the four chosen cohorts, again indicating the key role of the ECM in gastric cancer progression.

Considering the striking performance of the ECM among all the prognostic pathways, we extracted the gene set of “core matrisome” from MatrisomeDB, consisting of all the ECM-related genes, including those encoding 195 ECM glycoproteins, 44 ECM collagens, and 35 proteoglycans (**Supplementary Table S4**). These ECM-related genes were then used to cluster the meta-cohort into three groups named ECM cluster A, ECM cluster B, and ECM cluster C (**Figure 1F**); the ECM clustering shared great similarity with the pathway clusters (**Supplementary Table S5**; Kappa value = 0.69, p-value < 0.0001). As expected, the three ECM clusters displayed significant differences in survival (**Figure 1E**; log-rank test, p-value < 0.001). In summary, this evidence strongly supported the core matrisome as the major pathway in gastric cancer progression.

### Clinical and Different Biological Progress Traits of ECM Phenotypes in ACRG Cohort

**Figure 2:**
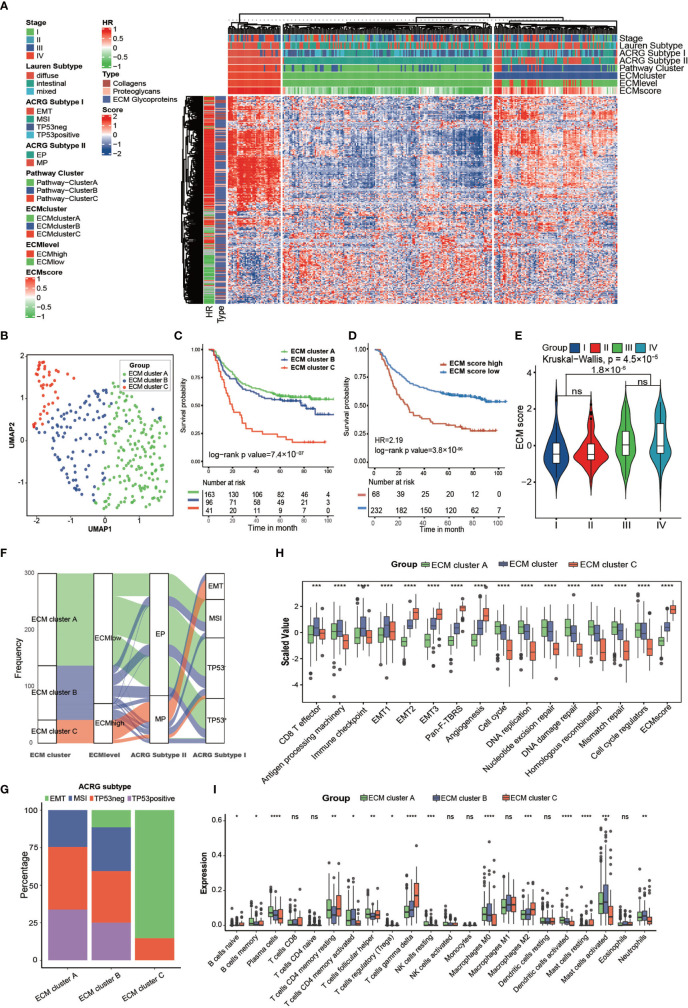
Clinical and transcriptome characteristics of ECM clusters in the ACRG cohort. **(A)** Heat map showing unsupervised clustering of 274 types of ECM for 300 patients in the ACRG cohort. Tumor stage, Lauren subtype, ACRG subtype I, ACRG subtype II, Pathway cluster, ECM level, and ECM score are shown as patient annotation. The hazard ratio and subtypes of ECM in the ACRG cohort are also shown in the ECM annotation. **(B)** A UMAP plot of 300 patients by dimensionality reduction of 274 types of ECM showing 3 distinct ECM clusters. **(C)** Kaplan–Meier curves for overall survival of 300 patients in the ACRG cohort with 3 distinct ECM clusters. The sample size of ECM clusters A, B, and C were n = 163, n =9 6, and n = 41, respectively. Log-rank test, p-value =7.4 × 10^−7^. **(D)** Kaplan–Meier curves for the ECM score by the best cut off value in the ACRG cohort. The numbers of patients in the ECM score high and ECM score low groups were n = 68 and n = 232, respectively. Log-rank test, p-value = 3.8 ×10^−6^. **(E)** Violin plot showing that the ECM scores are different among different tumor stages. Kruskal–Wallis test, p-value = 4.5 × 10^−5^. The ECM score of stage I and II is lower than that in stage III and IV, Student’s t test, p-value = 1.8 × 10^−6^. **(F)** Alluvial diagram showing the different ECM clusters in the different ECM levels, ACRG subtype I, and ACRG subtype II. **(G)** Stacked bar chart showing the proportion of ACRG subtypes in the different ECM clusters. **(H, I)** Different biological status and immune cell infiltration patterns of ECM clusters. The top and bottom of the boxes represent the interquartile range of the values. The thick lines in the middle of the boxes represent the median values. The black dots show the outliers. The statistical differences among different ECM clusters were evaluated using the Kruskal−Wallis test. Statistical p-value (*P < 0.05; **P < 0.01; ***P < 0.001, ****P < 0.0001). ns, not significant.

To explore the clinical and transcriptomic characterization of ECM phenotypes, we chose the ACRG cohort for further study. Similarly, we clustered the 300 samples into three pathway clusters with 147 common prognostic pathways and three ECM clusters with 274 ECM genes (**Figures 2A, B** and **Supplementary Figures S2A–E**). There was high consistency between the pathway clusters and the ECM clusters (**Supplementary Table S6**; kappa value = 0.68, p-value < 0.0001), and different ECM clusters showed great differences in OS and relapse free survival (RFS) (**Figure 2C**, log-rank test, p-value = 7.4 × 10^−7^; **Supplementary Figure S2H**, log-rank test, p-value = 7 × 10^−7^). The most enriched biological processes still shared great similarity with those in the meta-cohort (**Supplementary Figures S2F, G**).

The GSVA score of the “core matrisome” was used as the ECM score for the gene set that consisted of all the ECM genes and could reflect the ECM deposition status. We also tried to shorten this list to obtain a more precise list of ECM-related genes by taking the intersection of differential genes between ECM score high and the ECM score low group in the above four cohorts (**Supplementary Table S7**). However, the shortened gene list was less competent in predicting prognosis. The three ECM clusters had distinct ECM scores (**Supplementary Figure S2I**). As expected, the ECM score could predict OS (**Figure 2D**; HR = 2.19, log-rank test, p-value = 2.8 × 10^−6^) and RFS (**Supplementary Figure S2J**; HR = 2.47, log-rank test, p-value = 5.6 × 10^−7^). Tumors at stage III/IV had higher ECM scores than those of tumors at stage I/II (**Figure 2E**). Molecular subtypes analysis showed that most of ECM cluster C overlapped with the EMT subtype and none of ECM cluster A belonged to the EMT subtype. Additionally, a higher ECM score was highly associated with a mesenchymal phenotype and diffuse type of Lauren class (**Figures 2F, G** and **Supplementary Figure S2K**) (8). Activation of the EMT program could permit tumors to enter the cancer stem cell (CSC) state, which is resistant to most conventional therapeutics and the major reason for failure of eradicating carcinoma (30, 31). Thus, we deduced that ECM deposition constructed the niche for CSC, which could hinder the efficacy of multiple therapeutics.

The core matrisome turned out to be the major factor in gastric cancer progression; therefore, we characterized the ECM phenotypes with relevant biological processes involved in cancer progression (**Supplementary Table S8**) (32). The results showed that almost all chosen biological processes exhibited significant differences among the three ECM clusters (**Figure 2H**). The ECM score correlated positively with EMT-related processes and negatively with processes involved in DNA replication and DNA repair, which happened to be the feature of CSCs (**Supplementary Figure S2A** and **Supplementary Table S9**). We also analyzed the expression of transforming growth factor beta (TGFβ)-EMT pathway-related genes (*VIM*, *COL4A1*, *PDGFRA*, *SMAD9*, *TGFB2*, *TWIST1*, *ZEB2*, *CDH1*), DNA damage repair-related genes (*BRCA1*, *BRCA2*, *MLH1*, *MSH2*, *MGMT*, *APEX1*, *FEN1*), and immune checkpoint-related genes (*CD80*, *CD86*, *CTLA4*, *HAVCR2*, *IDO1*, *LAG3*, *PD1*, *PDL1*, *TIGIT*, *TNFRSF9*) in the ECM clusters of the ACRG cohort. The results were consistent with the related biological processes (**Supplementary Figures S3C–E**).

Additionally, the results indicated that different ECM phenotypes showed different immunocompetences. Therefore, we analyzed the immune infiltration pattern of the ECM phenotypes. ECM cluster C showed highest level of M2 and T gamma delta cells, which were identified risk factors for the OS of patients with gastric cancer, and lowest level of activated dendritic cells, M0 macrophages, activated mast cells, and neutrophils, which were identified as favorable factors for the OS of patients with gastric cancer (**Figure 2I**) (9).

### Clinical and Multi-Omics Traits of ECM Phenotypes in the TCGA Cohort

**Figure 3:**
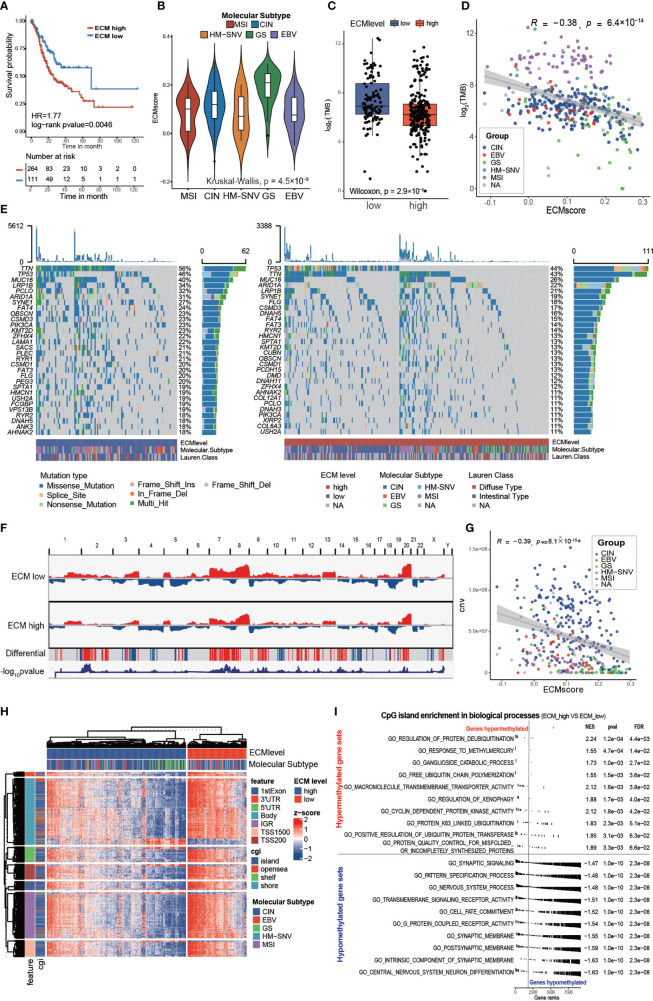
Characteristics of the ECM pattern in TCGA-STAD molecular subtypes and multi-omics level. **(A)** Kaplan–Meier curves for the ECM score by best cut off value in the TCGA-STAD cohort. The numbers of patients in the ECM score high and ECM score low groups were n = 264 and n = 111, respectively. Log-rank test, p-value = 0.0046. **(B)** Violin plot showing that the ECM scores are different among different molecular subtypes in the TCGA-STAD cohort. Kruskal−Wallis test, p-value = 4.5 × 10^−9^. MSI (n = 61), CIN (n = 207), HM-SNV (n = 7), GS (n = 45), EBV (n = 27). **(C)** Difference in the TMB between the ECM high and ECM low groups. The scattered dots indicate the TMB of each sample. The top and bottom of the boxes represent the interquartile range of the values. The thick lines in the middle of the boxes represent the median value. Wilcoxon rank sum test, p-value = 2.9 × 10^−7^. **(D)** Scatter plot depicting the correlation between the ECM score and the TMB. Spearman correlation analysis, R = −0.38, p-value = 6.4 × 10^−14^. The color of the dots represents the molecular subtypes annotated by the legend. **(E)** An Oncoprint showing the gene mutation map of the ECM high (right, red) and ECM low (left, blue) groups. Each column represents a patient and the barplot in the right of each group indicates the gene mutation frequency of each gene in the corresponding group. The barplot on the top shows the TMB. The gene mutation types are annotated in the legend. Molecular subtypes and Lauren subtypes are also shown as patient annotation. **(F)** CNV pattern of the ECM high and ECM low groups. The length of the plot represents the whole genome and each vertical line represents a gene; red for gain of copy number and blue for loss of copy number. The penultimate line mark the genes with differential copy number between the two groups; a red stripe for higher and a blue stripe for lower copy number in the ECM high group. The last line indicates the −log10 transformed chi-squared test p-value of the copy number difference. **(G)** Scatter plot depicting the correlation between the ECM score and CNV. Spearman correlation analysis, R = −0.39, P = 8.1 × 10^ −15^. The color of the dots represents the molecular subtypes annotated by the legend. **(H)** Heat map exhibiting the DNA methylation pattern of the ECM high and ECM low groups. The locations of each DNA methylation site are indicated in the left annotation. The molecular subtype is shown as patient annotation. **(I)** GSEA enrichment of CpG islands in biological processes between ECM high group and ECM low groups. The upper part shows the top10 hypermethylated biological processes and the lower part shows the top10 hypomethylated biological processes. The enrichment plot, normalized enrichment score (NES), p value and false discovery rate (FDR) are shown in the right.

Benefitting from multi-omics data, the TCGA cohort contains data related to constructed comprehensive molecular subtypes for gastric cancer, including genome stable (GS), microsatellite instability (MSI), EBV infection, and chromosomal instability (CIN). A higher ECM score was associated with the GS subtype and unfavorable prognosis, whereas a lower ECM score was associated with the EBV or MSI subtypes and favorable prognosis (**Figure 3A**, log-rank test, p-value = 0.0046; **Figure 3B**).

Gene instability, evaluated using the tumor mutation burden (TMB), would result in more neo-antigens, increasing the opportunity for immune recognition and clearance (33, 34). Besides, chemotherapeutic drugs function through damaging DNA integrity of rapidly cycling cancer cells (35). Thus, evaluation of the gene mutation load is very important for the precise administration of medication. First, we found that the high ECM score group had a lower TMB than the low ECM score group in the TCGA cohort (**Figure 3C**; Wilcoxon rank sum test, p-value = 2.9 × 10^−7^). In addition, the TMB correlated negatively with the ECM score (**Figure 3D**; Spearman correlation, r = −0.38, p-value = 6.4×10^−14^). Subgroup analysis showed that the correlation between ECM score and TMB differed in different molecular subtypes (**Supplementary Figures S4A–E**), which was highest in GS group (Spearman correlation, r = −0.5, p-value = 0.0014) and insignificant in MSI group (p>0.05). Furthermore, the low ECM score group presented a more extensive TMB than the high ECM score group for the levels of individual altered genes in the top 30 most frequently mutated genes (**Figure 3E**). According to **Figure 2H**, the ECM score correlated negatively with DNA replication, which might explain why a higher ECM score was associated with a lower TMB. Similarly, the high ECM score group tended to have less gain or loss in copy number and more wild-type genes (**Figure 3F**). In addition, the total CNV was also correlated negatively with the ECM score (**Figure 3G**; Spearman correlation, r= −0.39, p-value = 8.1×10^−15^), and the results in different molecular subtypes were about the same (**Supplementary Figures S4F–J**).

Epigenetic abnormalities are widespread among all tumor types, which also play an important role in drug resistance and immune surveillance (36–38). Therefore, we examined the association of the ECM score with DNA methylation. Interestingly, the high ECM score group had a lower level of DNA methylation in all DNA parts except for 3’ untranslated region, and the CpG island and CpG shore, which are associated with inhibiting gene expression, were enriched in the low ECM score group (**Figure 3H** and **Supplementary Figures S4K–L**). Furthermore, we compared the CpG island abundance between the high ECM score and the low ECM score group. GSEA enrichment results showed that, in the high ECM score group, the most hypomethylated biological processes were synapse development and cell differentiation, which may lead to EMT and cell stemness, and the most hypermethylated biological processes were less significant to be mentioned (**Figure 3I**). Global hypomethylation is an important feature of naïve pluripotent cells and complex regulation of the epigenome also promotes CSCs formation (39, 40). Therefore, we speculated that ECM deposition might promote CSC formation through an epigenetic mechanism.

### The ECM Score Predicts Chemotherapeutic Benefits

**Figure 4:**
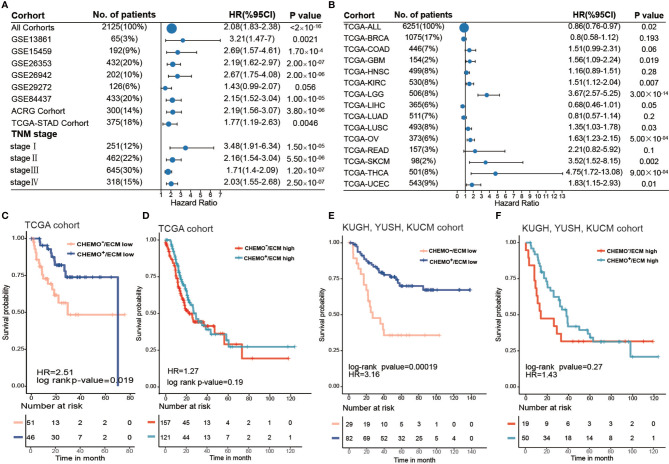
ECM score is a prognostic biomarker and could predict chemotherapy response. **(A)** Forest plot showing the difference in prognosis between ECM high and low groups in independent gastric cancer cohorts and different tumor stages. The horizontal coordinates represent the hazard ratio of the ECM high group relative to the ECM low group and the horizontal line represents the 95% confident interval of the hazard ratio. The size of the dot indicates the sample size of the independent group. **(B)** Forest plot showing the difference in prognosis between the ECM high and low groups in 14 types of solid tumors from the TCGA datasets. The horizontal coordinates represent the hazard ratio of the ECM high group relative to the ECM low group and the horizontal line represents the 95% confident interval of hazard ratio. The size of the dot indicates the sample size of the independent group. **(C)** Kaplan–Meier curves for overall survival of the ECM high group in the TCGA-STAD cohort grouped by chemotherapeutic history. CHEMO^-^/ECM high, n = 157; CHEMO^+^/ECM high, n = 121. Log-rank test, p-value = 0.19. **(D)** Kaplan–Meier curves for overall survival of the ECM low group in the TCGA-STAD cohort grouped by chemotherapeutic history. CHEMO^−^/ECM low, n = 51; CHEMO^+^/ECM low, n = 46. Log-rank test, p-value = 0.019. **(E)** Kaplan–Meier curves for overall survival of the ECM high group in the KUGH, YUSH, and KUCM cohorts grouped by chemotherapeutic history. CHEMO^−^/ECM high, n = 19; CHEMO^+^/ECM high, n = 50. Log-rank test, p-value = 0.27. **(F)** Kaplan–Meier curves for overall survival of the ECM low group in the KUGH, YUSH, KUCM cohort grouped by chemotherapeutic history. CHEMO^−^/ECM low, n = 29; CHEMO^+^/ECM low, n = 82. Log-rank test, p-value = 0.00019.

Upon dividing the specific data sets by the best cutoff value of the ECM score, significant differences in OS were observed between the low and high ECM score groups for all gastric cancer data sets except GSE29272 (HR, 1.43; 95% CI, 0.99–2.07) (**Figure 4A** and **Supplementary Table S10**). Meanwhile, the prognostic value of the ECM score was also validated in five other independent data sets (GSE13861: HR, 3,21; 95% CI, 1.47–7.0; GSE15459: HR, 2.69; 95% CI, 1.57–4.6; GSE26253: HR, 2.19; 95% CI, 1.62-2.97; GSE26942: HR, 2.67; 95% CI, 1.75–4.08; GSE84437: HR, 2.15; 95% CI, 1.52–3.04; **Supplementary Figures S5C–G**). Moreover, the ECM score could also predict poor prognosis in each stage of gastric cancer (stage I: HR, 3.48; 95% CI, 1.91–6.34; stage II: HR, 2.16; 95% CI, 1.54–3.04; stage III: HR, 1.71; 95% CI, 1.40–2.09; and stage IV: HR, 2.03; 95% CI, 1.55–2.68). These results suggested that the ECM score could be prognostic factor that is independent of tumor stage in gastric cancer (**Supplementary Figures S5A, B**).

Next, we investigated the performance of the ECM score in pan-cancer. We evaluated the predictive value of the ECM score for 14 types of solid tumors in the TCGA cancer cohort, comprising 6251 samples in total (**Supplementary Table S11**). The results showed that the ECM score was a risk factors for eight types of cancer in the TCGA cohorts, including thyroid cancer, brain lower grade glioma, skin cutaneous melanoma, uterine corpus endometrial carcinoma, ovarian serous cystadenocarcinoma, glioblastoma, kidney renal clear cell carcinoma, and lung squamous cell carcinoma, but was irrelevant to other types of cancer, which indicated the biological heterogeneity of the ECM among distinct cancer types (**Figure 4B**).

Chemotherapy is a crucial treatment to supplement surgery in patients with gastric cancer. However, currently, there is no biomarker that can effectively predict a patient’s chemotherapy response and even guide the choice of chemotherapeutic regimens. Our results demonstrated that a higher ECM score was associated with the EMT molecular subtype in the ACRG cohort and with the GS molecular subtype in the TCGA-STAD cohort, which were tolerant to chemotherapy. To explore the capacity of the ECM score to predict the chemotherapy response, we first evaluated the best cut off value of the ECM score for patients in the TCGA-STAD cohort who had received chemotherapy according to their prognosis, which could divide the TCGA-STAD cohort into ECM low and ECM high groups. Combined with the chemotherapeutic history, the best cut off value further stratified the TCGA-STAD cohort into Chemo^+^/ECM high, Chemo^−^/ECM high, Chemo^+^/ECM low, Chemo^−^/ECM score low groups. Interestingly, survival analysis indicated that patients with a low ECM score could benefit from chemotherapy (**Figure 4C**; log-rank test, p-value = 0.019), while there was no significant difference between the chemotherapy and non-chemotherapy group even when the sample size was larger (**Figure 4D**; log-rank test, p-value > 0.05). Besides, more patients with a low ECM score showed a complete response to chemotherapy and less progressive disease compared with patients with a high ECM score (**Supplementary Figure S5H**). To further verify this result, stage II, III, or IV gastric cancer without distant metastasis (n = 180) in three cohorts [GSE26899 for the KUGH cohort, GSE26901 for the KUCM cohort, and GSE13861 for the YUSH cohort; (**Supplementary Table S12**)] with complete chemotherapy information were integrated for survival analysis (log-rank test, p-value = 6.5 × 10^-6^) Likewise, patients with a low ECM score could achieve a satisfactory chemotherapy response (**Figure 4E**; log-rank p-value = 0.00019), while patients with a high ECM score could not (**Figure 4F**; log-rank test, p-value > 0.05). Our results strongly supported the view that the ECM score could predict poor prognosis and the response to chemotherapy.

### *In Vitro* Study Indicated That the ECM Could Influence the Invasion and Chemoresistance of Gastric Cancer Cells

**Figure 5:**
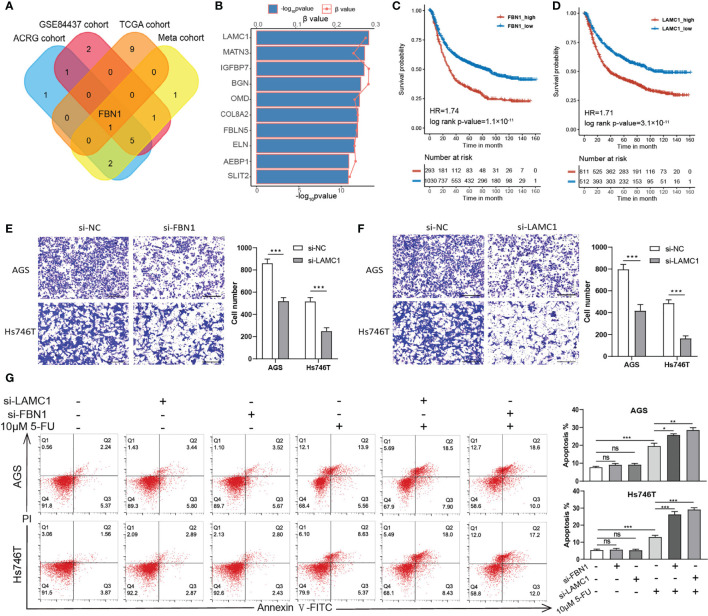
ECM could influence the invasion and drug tolerance of gastric cancer cells. **(A)** A Venn diagram showing the intersection of top 10 hub ECM genes of four cohorts (TCGA-STAD, ACRG, GSE84437 and meta-cohort). *FBN1* is the only common hub ECM gene in the four cohorts. **(B)** Univariate Cox regression results of all ECM in the meta cohort. The top 10 ECM genes with the lowest p values are shown. The horizontal bars shows the −log_10_ p-value of the univariate Cox regression and the horizontal coordinates of the dots show the β value of univariate Cox regression. **(C, D)** Kaplan–Meier curves for *FBN1* and *LAMC1* by best cut off value in the meta cohort. *FBN1*: HR, 1.74, Log-rank p-value = 1.1 × 10^−11^. *LAMC1*: HR, 1.71, Log-rank p-value = 3.1 × 10^−11^. **(E, F)** Transwell invasion assay performed in AGS and Hs746T cells transfected with control siRNA, *FBN1* siRNA, or *LAMC1* siRNA. **(G)** Apoptosis was determined using fluorescence activated cell sorting (FACS) analysis by Annexin V-FITC and propidium iodide (PI) co-staining (left panel), and Annexin V^+^ cell populations were defined as apoptotic (right panel). Statistical p-value (*P < 0.05; **P < 0.01; ***P < 0.001; ns, not significant).

To further verify our analysis, we chose two representative ECM genes for the *in vitro* experiment. Firstly, we conducted Spearman correlation analysis for two ECM genes according to their expression levels in the TCGA-STAD, ACRG/GSE66229, GSE84437, and the meta-cohort and submitted the correlation networks to Cytoscape to find a hub gene for ECM deposition (**Supplementary Figures S6A–E** and **Supplementary Table S13**). *FBN1* was identified as the only common top 10 hub gene among all the cohorts. Fibrillin 1 serves as scaffold for elastic fibers and as a reservoir for growth factors like TGFβ (**Figure 5A**) (41, 42). Then, we performed univariate Cox regression for all the ECM genes in the meta-cohort and identified *LAMC1*, which encodes laminin subunit gamma 1, an essential component of the basement membrane that is involved in multiple types of cancer progression (43–46), as the risk factor with the lowest p-value (**Figure 5B**). Both genes correlated significantly with poor prognosis in the meta-cohort (**Figures 5C, D**; *LAMC1*: HR = 1.71, log-rank test, p-value < 0.0001; *FBN1*: HR = 1.74, log-rank test, p-value < 0.0001). After knocking down the expression of *LAMC1* and *FBN1* separately in Hs746T cells, a metastatic and mesenchymal like cell line, the invasiveness of Hs746T cells was compromised significantly. Similar but less conspicuous results were when the experiment was repeated in AGS cells, a primary and epithelial like cell line (**Figures 5E, F**). We speculated that relative lower expression of the targeted genes in AGS cells accounted for the less significant influence of treatment. However, it is plausible to claim that the ECM could have impact on the EMT phenotype. To check whether the ECM could influence the chemotherapeutic response, we treated Hs746T and AGS cells with 10 μM 5 fluorouracil (5FU), a first-line chemotherapy drug, (1) combined with *LAMC1* or *FBN1* knockdown for 48 h. Knocking down *LAMC1* or *FBN1* did not influence the survival of Hs746T or AGS cells. However, knocking down *LAMC1* or *FBN1*, or both could sensitize Hs746T cells to 5-FU; the same phenomenon was observed in AGS cells but with less significance. In addition, the mesenchymal-like Hs746T cell line was more tolerant to 5-FU than the epithelial-like AGS cell line (**Figure 5G**).

## Discussion

Growing evidence suggests that the ECM is an indispensable but enigmatic component of the tumor microenvironment (13, 15). Aberrant constitution of the ECM is involved in all the cellular processes throughout cancer initiation, progression, and dissemination, and, in most cases, correlated with more aggressive tumors and poorer prognosis (19). In breast cancer, researchers found that decellularized ECM from tumor-bearing and obese mammary glands drives triple-negative breast cancer (TNBC) cell invasion, and collagen VI was found to be the driver protein by proteomic analysis (47). In colon cancer, Romero-López et al. (48) extracted and compared ECM from normal human colon and colon tumor that had metastasized to liver and even seeded tumor cells in these ECM. The results showed that cells seeded in tumor ECM had higher levels of free NADH along with glycolytic rate and more capable of inducing tumor-like vasculature compared with those seeded in normal ECM. Contradictorily, in pancreatic ductal adenocarcinoma, decreasing ECM with an anti-LOXL2 antibody *in vivo* boosted tumor growth and diminished overall survival, suggesting a protective role of ECM (20). Also, in melanoma, there is evidence that aging fibroblasts were less capable of secreting ECM, especially HAPLN1, resulting in a more aligned ECM that promoted metastasis of melanoma cells (49). Hence, it seems that the ECM also share heterogeneity among different tumor types and the interplay between ECM and tumor cells is still intricate.

Additionally, the ECM is also an interference factor during anti-tumor therapy. Excessive ECM deposition and stiffening in solid tumors could also induce physical and biological barriers for chemotherapy, a major problem faced by current cancer research. For example, ECM deposition in liver metastasis of colorectal tumor could enhance angiogenesis and anti-angiogenic therapy resistance, while inhibiting ECM deposition with drugs targeting the renin-angiotensin system could reverse resistance to anti-angiogenic bevacizumab (22). Also, decreasing ECM stiffness with lysyl oxidase (LOX) inhibitors increased drug penetration and overcame chemotherapy resistance in triple negative breast cancer (50). However, to date, the clinical and multi-omics characterization of the ECM in gastric cancer and the potential of the ECM to predict prognosis and chemotherapy response had not yet been systematically explored, neither did any clinical trials investigate the role of ECM deposition in anti-tumor therapy resistance.

Exploiting GSVA algorithms, we scored all the gene set canonical pathways and ECM was identified as core factor in gastric cancer progression with highest degree among all prognostic pathways in the overlap of four cohort. Then, we resolved the ECM constitution pattern and depicted the overall landscape of the clinical and multi-omics characterization of the ECM in gastric cancer. Integrated analysis detected that the ECM score performed well to predict the prognosis and chemotherapy response in gastric cancer. The ECM score was a robust risk factor in different stage of gastric cancer and in different cohorts. It was verified in both the TCGA-STAD cohort and the KUGH, YUSH, and KUCM cohorts that chemotherapy showed a poor effect in patients with gastric cancer with a high ECM score, the result of which might be instructional for precision medicine.

In the ACRG cohort, the ECM score was exclusively high in patients with the EMT molecular subtype. Previous studies had identified EMT or a mesenchymal phenotype as predicators for poor prognosis and resistance to anti-cancer drug therapy in multiple cancer types (8, 30, 51–53). The EMT-like change could also enable cancer cells to acquire a cancer stem cell phenotype, which has received unanimous acceptance as the backbone of drug resistance (30). Counterintuitively, our results showed that cancers with a higher ECM score showed a remarkably low level of proliferation activity, which might be ascribed to the dominant state of CSCs. There is considerable evidence demonstrating that ECM remodeling could be the upstream signal that regulates the EMT or CSCs phenotype through a mechanochemical pathway in cancer cells (15, 54–56)

In the TCGA-STAD cohort, the ECM score was exclusively high in patients of the GS molecular subtype. The GS molecular subtype is characterized by a low TMB. Most chemotherapy imposes DNA damage on rapidly proliferating cancer cells that lack adequate DNA repair (35). Hence, we deduced that gene stability and relatively slow DNA replication could restrain the effectiveness of chemotherapeutic drugs, which happened to be the feature of those cancers with a high ECM score. Our results demonstrated that the ECM score was closely related to the TMB as well as CNV, further explaining its capacity to predict drug response.

The role of non-genetic or epigenetic mechanisms to regulate drug resistance is vital but poorly understood. Aberrant epigenetic regulation is common among all tumor types and has long been considered as a regulator of drug resistance, and several epigenetic therapies have been involved in preclinical trials (57–60). Our data indicated that ECM deposition in gastric cancer might alter epigenetic states, thus influencing the drug response. Determining the interconnection between ECM remodeling and epigenetic alteration would deepen our understanding of drug-tolerant cancer.

Increased use of immunotherapy has revealed the presence of immune tolerance (61–64). It is not surprising that in accordance with our results, the tumor-associated ECM could also have immune modulatory effects and could regulate the migration and localization of immune cells (65, 66). Actually, combined treatment targeting both the immune and stroma microenvironment could lead to remarkable therapeutic effects (67, 68). Finally, we chose two representative ECM genes, *LAMC1* and *FBN1*, to further verify our findings. Knocking down these two genes impaired the invasion ability of cancer cells and sensitized cancer cells to chemotherapeutic drugs, which, to some extent, corroborated our analyses.

In short, in the current study, gene expression analysis identified ECM as the driving factor involved in gastric cancer progression. Hence, we systematically discussed the landscape of clinical, biological, and multi-omics characterization of the ECM constitution pattern in gastric cancer and found a higher ECM score is tightly associated with an epithelial to mesenchyme transition (EMT) phenotype, a gene stable (GS) molecular subtype, markedly lower somatic mutation rates, and a lower level of DNA methylation. In addition, the ECM score was identified as robust prognostic biomarker and predictive factor for the response to chemotherapy resistance, which was further verified experimentally. Our findings imply that ECM may foster chemotherapy resistance in gastric cancer genetically and epigenetically. Further investigation would help to solve the enigma of chemoresistance acquisition. The establishment of ECM score could also help to design personalized and precise chemotherapy and provide inspiration to develop combination therapy. Nevertheless, our study has some limitations as well. Detailed information regarding the treatment history of the enrolled patients with gastric cancer was inadequate, such as the prescription and duration of the chemotherapy, and the receipt of any other treatment, which would interfere with the precise identification of the best cut off point. Further treatment information gathering would help to refine the prediction model. Additionally, tumor heterogeneity is the main cause of chemotherapy tolerance, which means that sequencing of mixed tumor tissues might inevitably lead to bias. Standardized and sub-regional sample collection and, if conditional, single cell sequencing, would ensure an in-depth exploration of the role of the ECM in chemoresistance.

## Data Availability Statement

datasets presented in this study can be found in online repositories. The names of the repository/repositories and accession number(s) can be found in the article/**Supplementary Material**.

## Author Contributions

Conception and design of the study: XX, JC, and WG. Data collection and preprocessing: ML and XZ. Analysis and interpretation of data: ZY and FX. Manuscript writing: ZY, ML, and XZ. Study selection, *in vitro* experiment and statistical expertise: CW, EX, XW, LZ, HY, CR, HW, and YW. All authors contributed to the article and approved the submitted version.

## Funding

This work was supported by the Nanjing Medical Science and Technology Development [No. YKK16117], the Nanjing Health Science and Technology Development Special Fund for Distinguished Young Scholar [No. JQX19001], the Key Research Plan and Social Development Project of Jiangsu Province, China [No. BE2016603], and the National Ministry of Science and Technology Project [No. 2016YFC0104105].

## Conflict of Interest

The authors declare that the research was conducted in the absence of any commercial or financial relationships that could be construed as a potential conflict of interest.

## Publisher’s Note

All claims expressed in this article are solely those of the authors and do not necessarily represent those of their affiliated organizations, or those of the publisher, the editors and the reviewers. Any product that may be evaluated in this article, or claim that may be made by its manufacturer, is not guaranteed or endorsed by the publisher.
