# Supplementary material for: Extracellular Matrix Characterization in Gastric Cancer Helps to Predict Prognosis and Chemotherapy Response
Source: Front Oncol. 2021 Sep 27;11:753330. doi: 10.3389/fonc.2021.753330 (PMC8503650; doi:10.3389/fonc.2021.753330)
Supplement: Supplementary file 1 [file DataSheet_1.pdf]

Supplementary Figure S1. Overview of study design and identification of core matrisome as driving factor

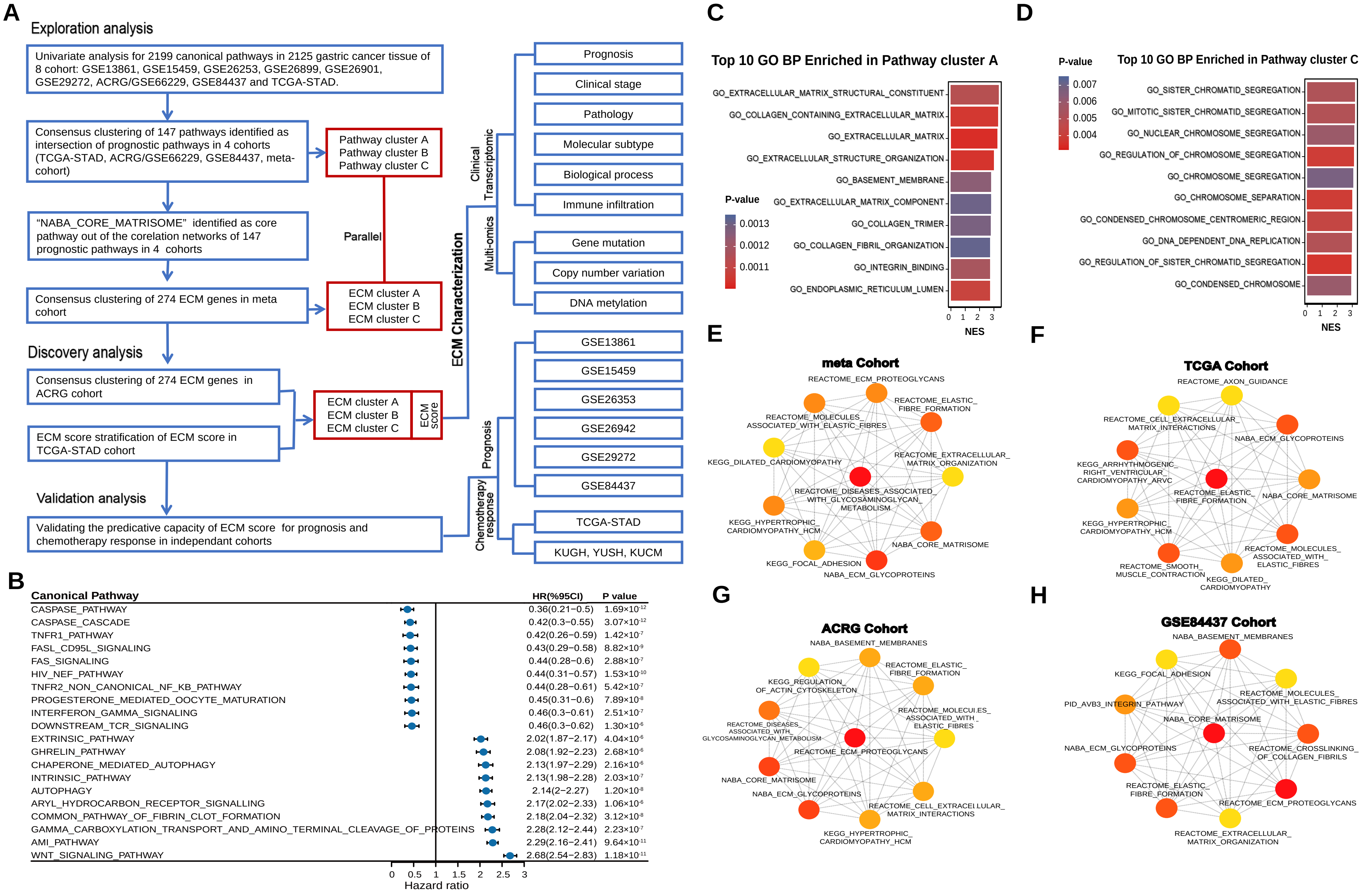

A, Overview of of the study. **B**, Top10 favorable and risk pathways in the meta-cohort estimated by univariate cox regression. **C** and **D**, GSEA results showing top10 biological processes enriched in pathway cluster A and C. NES, normalized enrichent score. **E-H**, Top10 prognostic hub pathways of meta-cohort, TCGA-STAD cohort, ACRG cohort and GSE84437 cohort.

Figure S2. Unsupervised clustering of ECM in ACRG cohort

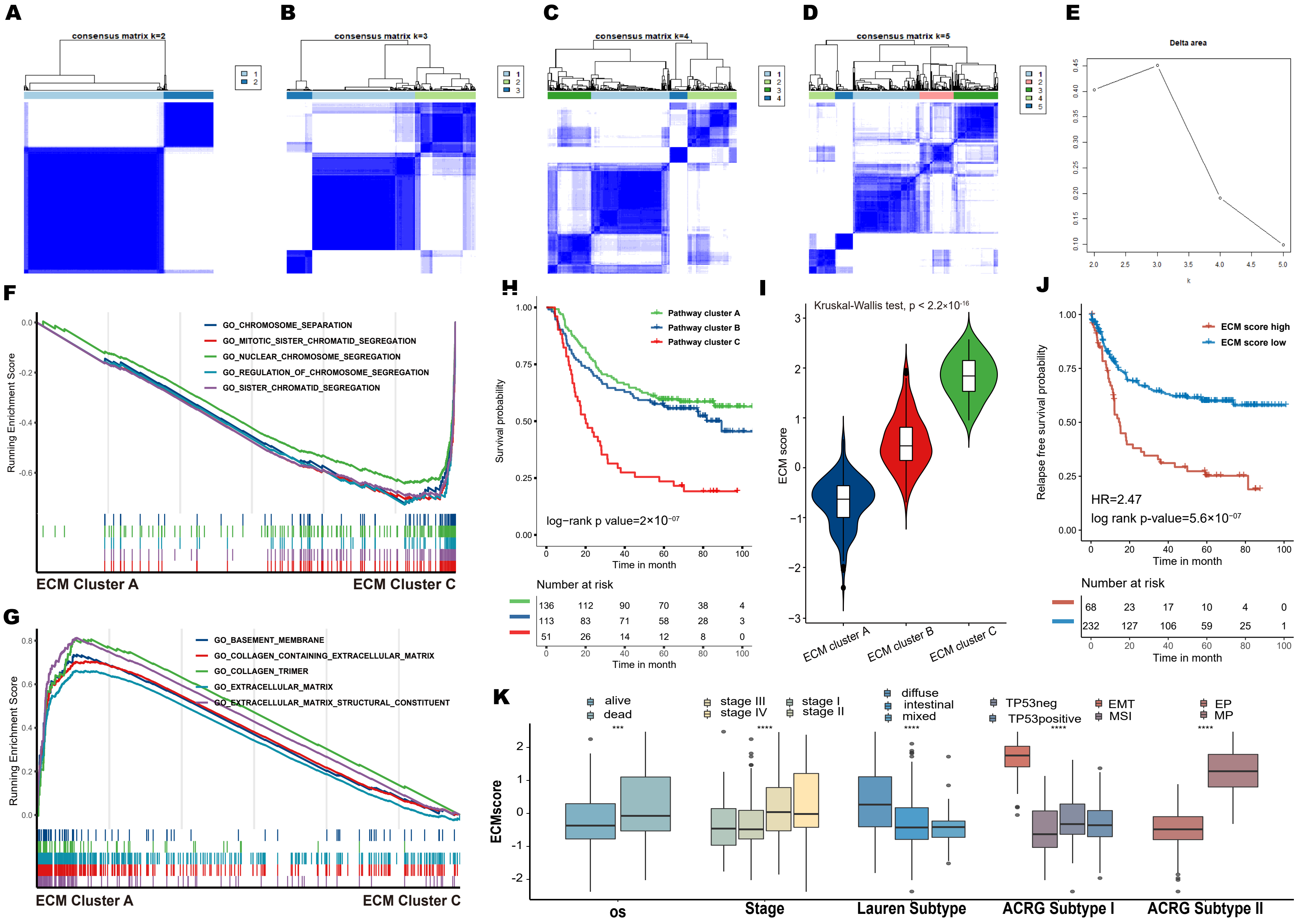

A-D, Consensus matrixes of the ACRG cohort for k = 2 - 5. E, Optical clustering chosen by delta area. **F and G**, GSEA running plot showing the biological process enriched in ECM cluster A and C. **H**, Kaplan−Meier curves for disease free survival of 300 patients in ACRG cohort with 3 distinct ECM clusters. log-rank test, p-value=7×10^{-7}. **I**, Violin plot showing the ECM score of 3 ECM clusters. Kruskal−Wallis test, $p < 2.2\times10^{-16}$. **J**, Kaplan−Meier curves for disease free survival of 300 patients in the ACRG cohort stratified by best cut off value of ECM score. Log-rank test, p-value=5.6×10^{-7}. **K**, Boxplot of the ECM score in different clinical subgroup.

Supplementary Figure S3. Correlation between ECM pattern and transcriptomic gene signatures

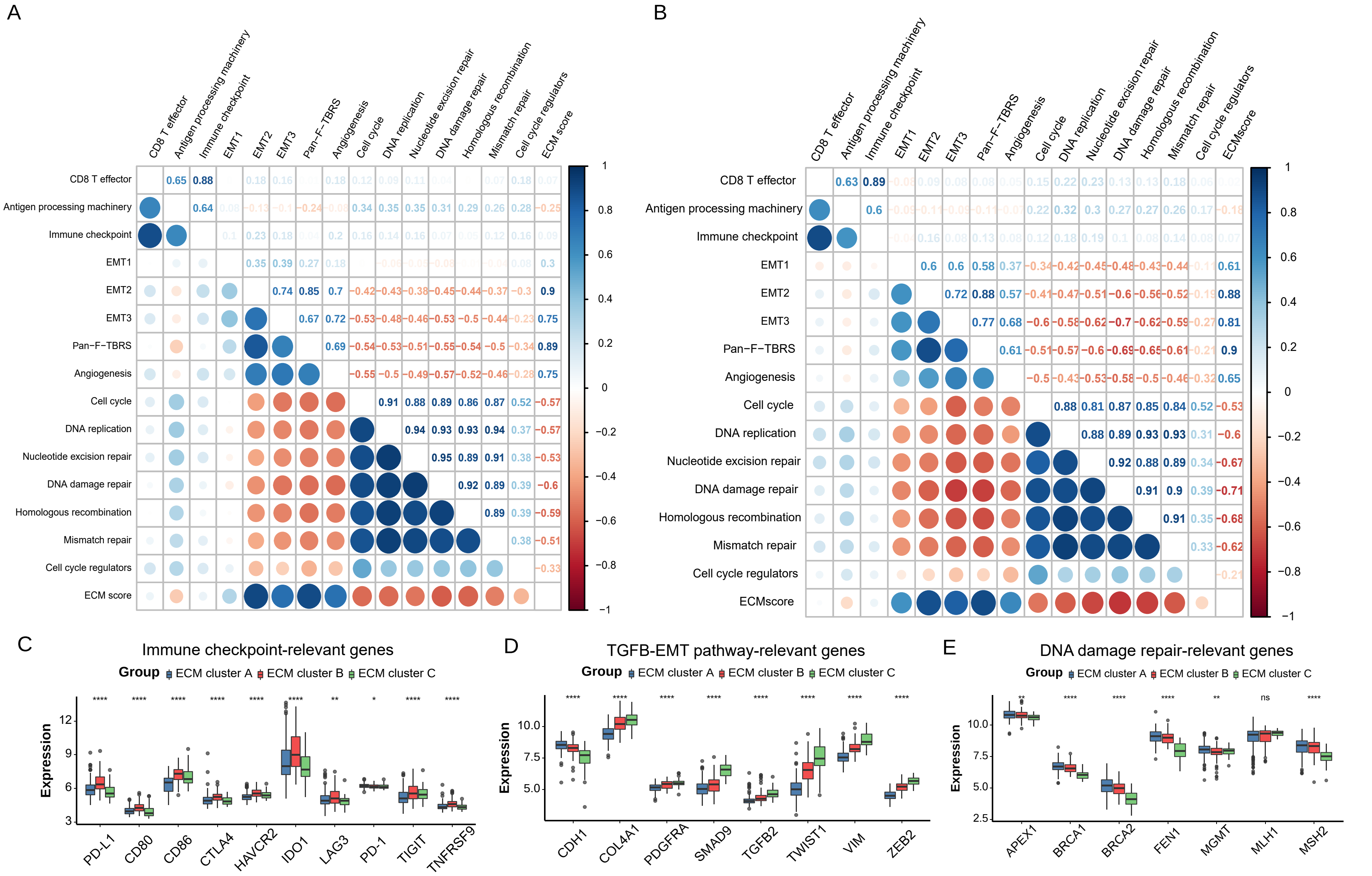

A and B, Correlations between the ECM score and the GSVA score of epithelial-mesenchymal transition (EMT), immune checkpoint, mismatch repair, and immune activation-related gene sets in the ACRG cohort (D) and the TCGA-STAD cohort (E) **C-E**, .Expression of the immune checkpoint-relevant, TGFB-EMT pathway-relevant and DNA damage repair-relevant genes in ECM clusters of the ACRG cohort.

Supplementary Figure S4. Stratification analysis of the correlation between ECMscore and TMB, CNV or DNA methylation

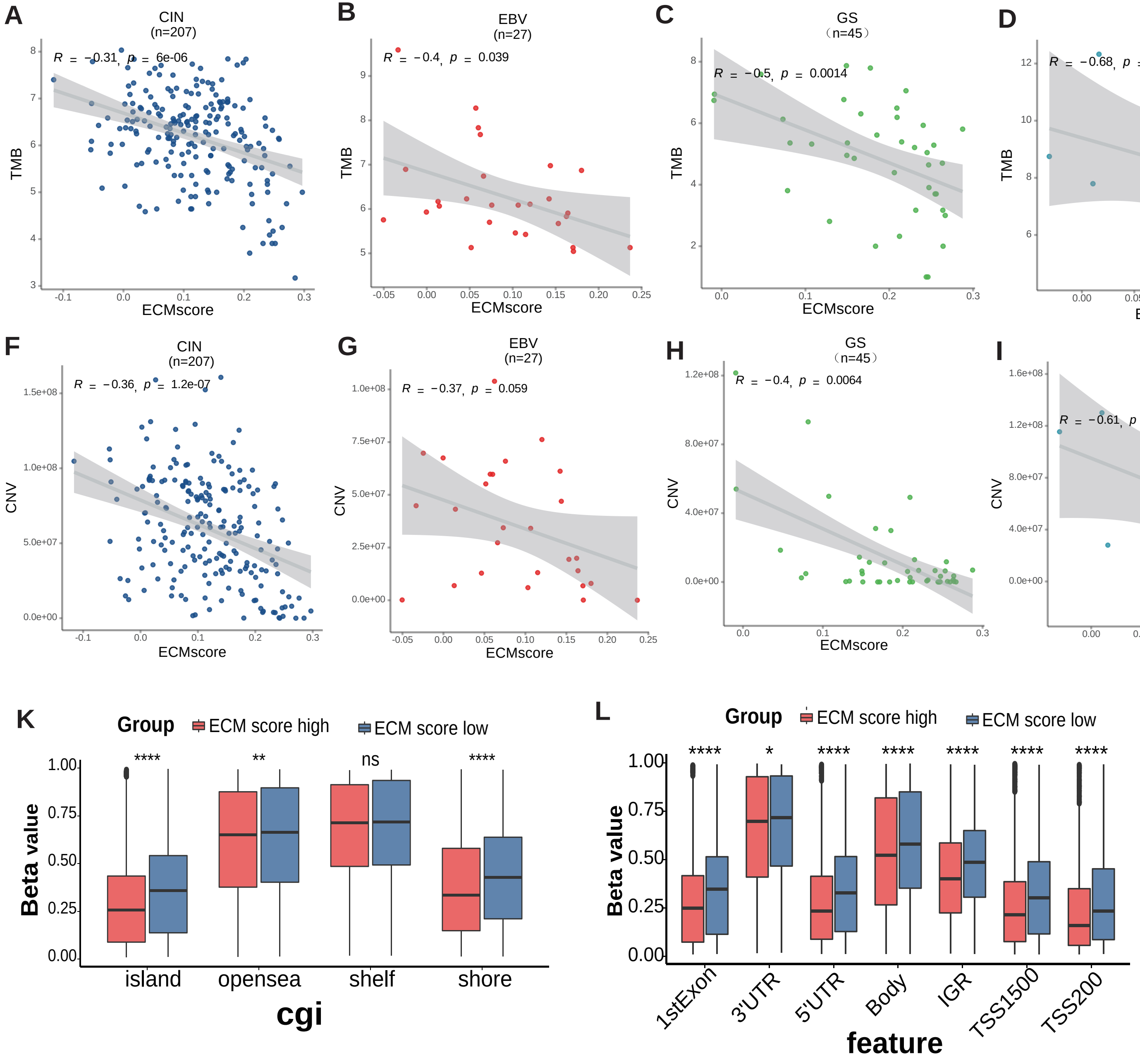

(A)-(E), Correlations between ECMscore and TMB in five different molecular subtypes in TCGA-STAD cohort. **(F)-(J),** Correlations between ECMscore and CNV in five different molecular subtypes in TCGA-STAD cohort. **(K)** and **(L)**, Methylation difference between the ECM high and low groups in different CGI coordinates and feature types.

Supplementary Figure S5. Clinical significance of ECM score in independent cohort

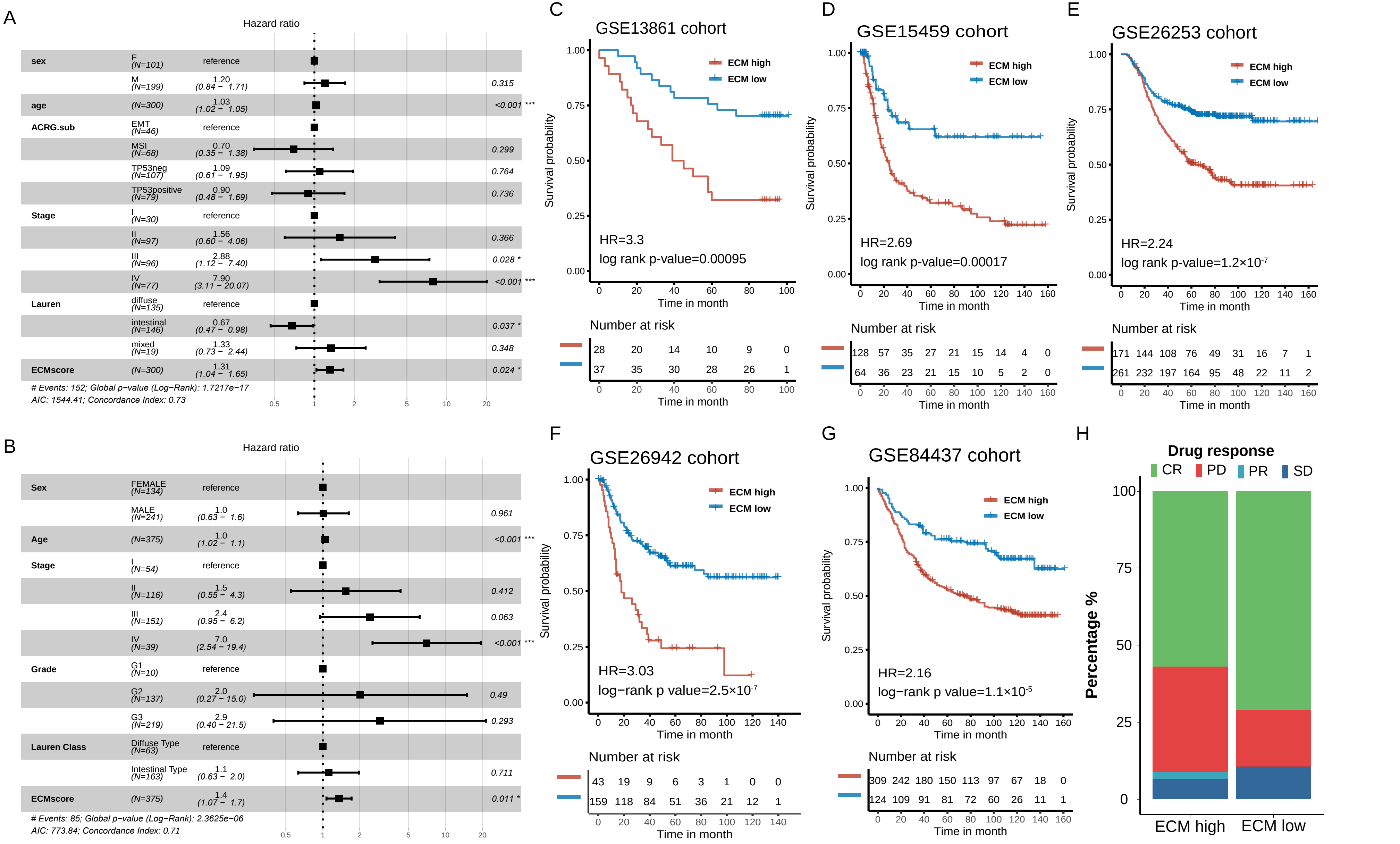

A and **B,** Forest plot showing multivariate regression result in the ACRG (A) and the TCGA-STAD (B) cohort. **C-G**, Kaplan-Meier curves for overall survival of independent cohorts (GSE13861, GSE15459, GSE26253, GSE26942, GSE84437 cohort) stratified by best cut off value of the ECM score. **H**, Stacked bar chart showing the proportion of different chemotherapy responses in the ECM high and low groups in the TCGA-STAD cohort.

Supplementary Figure S6. Core ECM scanning

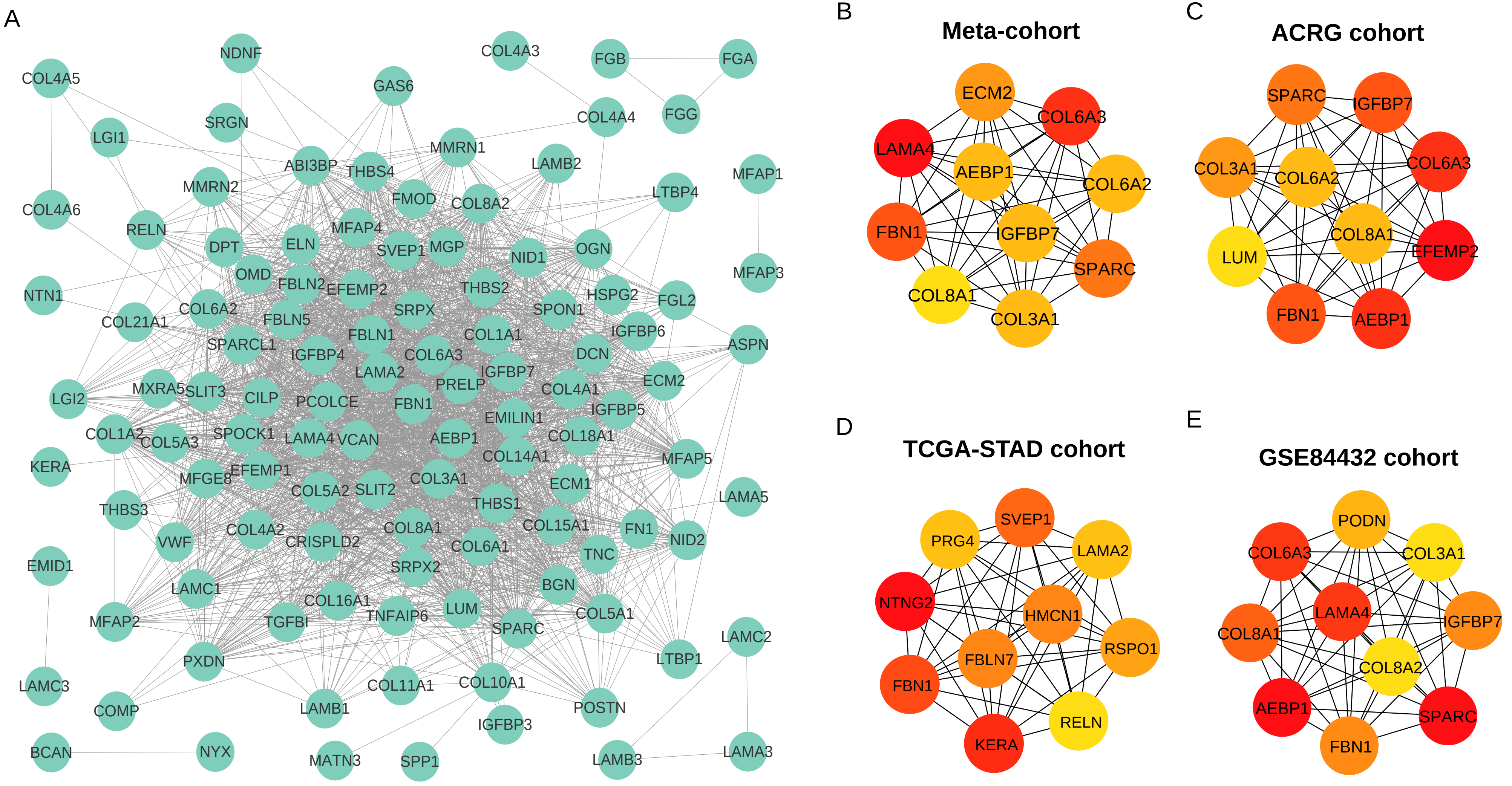

A, Correlation network of ECM in the meta-cohort. **B-E**, Top10 hub ECM genes in the meta-cohort, ACRG cohort, TCGA-STAD cohort and GSE84437 cohort.
